# Identification of *Asparagopsis armata*‐associated bacteria and characterization of their bioactive potential

**DOI:** 10.1002/mbo3.824

**Published:** 2019-04-29

**Authors:** André Horta, Celso Alves, Susete Pinteus, Cláudia Lopes, Nádia Fino, Joana Silva, Joana Ribeiro, Daniel Rodrigues, João Francisco, Américo Rodrigues, Rui Pedrosa

**Affiliations:** ^1^ MARE – Marine and Environmental Sciences Centre ESTM Polytechnic Institute of Leiria Peniche Portugal

**Keywords:** Algae, Antimicrobial, Antitumor, Epiphytic bacteria, Marine compounds, Marine symbiosis

## Abstract

Macroalgae‐associated bacteria have already proved to be an interesting source of compounds with therapeutic potential. Accordingly, the main aim of this study was to characterize *Asparagopsis armata*‐associated bacteria community and evaluate their capacity to produce substances with antitumor and antimicrobial potential. Bacteria were selected according to their phenotype and isolated by the streak plate technique. The identification was carried out by the RNA ribosomal 16s gene amplification through PCR techniques. The antimicrobial activities were evaluated against seven microorganisms (*Escherichia coli*,* Pseudomonas aeruginosa*,* Bacillus subtilis*,* Salmonella enteritidis*,* Staphylococcus aureus*,* Saccharomyces cerevisiae*,* Candida albicans*) by following their growth through spectrophotometric readings. Antitumor activities were evaluated in vitro on human cell lines derived from hepatocellular (HepG‐2) and breast carcinoma (MCF‐7) using the MTT method. The present work identified a total of 21 bacteria belonging to the genus *Vibrio*,* Staphylococcus*,* Shewanella*,* Alteromonadaceae*,* Bacillus*,* Cobetia*, and *Photobacterium*, with *Vibrio* being the most abundant (42.86%). The extract of S*hewanella* sp. ASP 26 bacterial strain induced the highest antimicrobial activity, namely against *Bacillus subtilis* and *Staphylococcus aureus* with an IC_50_ of 151.1 and 346.8 μg/mL, respectively. These bacteria (*Shewanella* sp.) were also the ones with highest antitumor potential, demonstrating antiproliferative activity on HepG‐2 cells. *Asparagopsis armata*‐associated bacteria revealed to be a potential source of compounds with antitumor and antibacterial activity.

## Graphical abstract

Macroalgae‐associated bacteria have showed to be an interesting source of compounds with therapeutic potential. In this study, *Asparagopsis armata*‐associated bacteria reveal to be a potential source of compounds with antitumor and antibacterial activity.

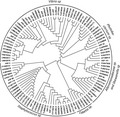

## 1 INTRODUCTION

Although many efforts are continuously being done for human's well‐being improvement, and life expectancy increase, some diseases continue to challenge medical sciences, such as infectious diseases due to microbial resistance to antimicrobial drugs, and cancer, which still lacks efficient treatments (Bray et al., 2018; Zheng, Sun, & Simeonov, 2018). In fact, recent statistics pointing out that, in 2050, infectious diseases will be the first cause of death all over the world, followed by cancer (O'Neill, 2018). The overuse and misuse of antimicrobial drugs have resulted in a continuous rise of antimicrobial‐resistant bacteria, against which most of the known antimicrobials are no longer effective (Luepke et al., 2017; Penesyan, Marshall‐Jones, Holmstrom, Kjelleberg, & Egan, 2009). As for cancer, the occurrence of tumor resistance to the available chemotherapeutics is also a major cause of concern. Tumor cells can present different responses to chemotherapies due to the heterogeneity of tumor mass. The same tumor can contain drug‐sensitive cells and resistant cells; consequently, chemotherapy treatments will only produce effects against drug‐sensitive cells, resulting in an ineffective treatment. Some cancers are also characterized by the presence of stem cells which promote tumor recurrence and chemotherapeutic resistance to standard treatments (Alves et al., 2018; Stavrovskaya, 2000; Zahreddine & Borden, 2013). As a result, infectious diseases and cancer represent major threats to public health, and consequently, there is an urgent demand for new active structures for the development of novel therapeutic agents.

Natural products have revealed to be the major source of compounds for drug development, and consequently, a large number of plants, animals, bacteria, and fungi have been examined for drug discovery (Villarreal‐Gomez, Soria‐Mercado, Guerra‐Rivas, & Ayala‐Sanchez, 2010). Due to the high complexity of marine ecosystem, the possibilities in this environment are unlimited, and therefore, in the last decades, scientists have focused on marine organisms as source of new bioactive molecules for the development of new chemical compounds (Blunt et al., 2018; Blunt, Copp, Munro, Northcote, & Prinsep, 2010, 2011; Jha & Zi‐rong, 2004; Penesyan, Kjelleberg, & Egan, 2010; Proksch, Edrada‐Ebel, & Ebel, 2003). Due to the extraordinary complexity of the marine environment, throughout evolution marine organisms established close associations with other species to ensure their survival. Many of these species are known to produce bioactive compounds through their secondary metabolism. However, it is now known that many of the bioactive compounds previously attributed to some invertebrates such as seaweeds and sponges were in fact produced or metabolized by their associated microorganisms (Goecke, Labes, Wiese, & Imhoff, 2012; Soria‐Mercado, Villarreal‐Gómez, Rivas, & Sánchez, 2012; Zheng, Han, Chen, Lin, & Yan, 2005). These compounds are normally produced as response to environmental and ecological challenges and many have already shown potent biological activities with high significance for biotechnological and pharmaceutical applications (Blunt, Copp, Keyzers, Munro, & Prinsep, 2012; Qiao et al., 2010). In this context, *Asparagopsis armata*, a red macroalgae, is already known to produce bioactive compounds with antitumor and antimicrobial potential (Alves, Pinteus, Horta, & Pedrosa, 2016; Alves, Pinteus, Rodrigues, Horta, & Pedrosa, 2018; Genovese, Tedone, Hamann, & Morabito, 2009); however, the biodiversity of its associated bacteria and their potential as source of new bioactive compounds have not yet been explored. Thus, the main aim of this study was the isolation and identification of *Asparagopsis armata*‐associated bacteria and the evaluation of their capacity to produce antitumor and antimicrobial substances.

## 2 EXPERIMENTAL PROCEDURES

### 2.1 Isolation and purification of associated marine bacteria

**Figure 1:**
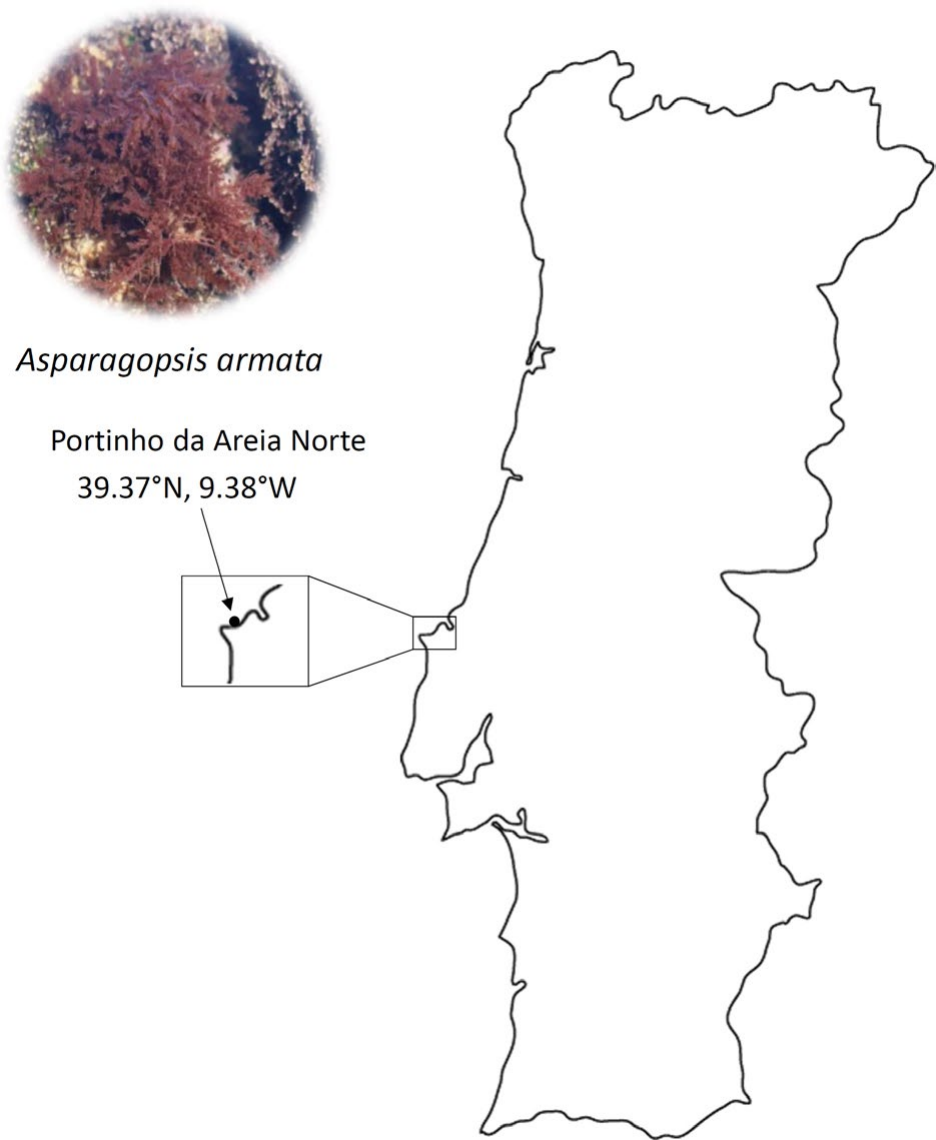
Sampling collection of *Asparagopsis armata* in Portinho da Areia Norte (39.37°N, 9.38°W), Peniche, Portugal

*Asparagopsis armata* seaweed was collected from Portinho da Areia Norte beach (39.37°N, 9.38°W), Peniche, Portugal (Figure 1) and immediately transported in sterile seawater to the MARE‐IPLeiria facilities. Samples were sequentially rinsed with sterile seawater to remove debris, epiphytes, and loosely attached bacteria. Following, seaweed samples were swabbed with sterile swabs to directly inoculate Petri dishes with marine agar. Plates were then incubated at room temperature (22°C) for 3–7 days. Colonies with distinct morphology were selected for further purification through streak technique.

### 2.2 Bacterial DNA extraction and genus identification

Isolated colonies were transferred to 10 mL of liquid Marine Broth (peptone 5 g, yeast extract 1 g, FePO_4_ 0.1 g, dissolved in 1 L seawater, pH 7.2–7.6) and incubated at room temperature until saturation. Bacterial suspension was then centrifuged and the pellets used for DNA extraction with the GeneJET^™^ Genomic DNA Purification Kit (Thermo Fisher Scientific, Vilnius, Lithuania), according to the manufacturer's instructions. Genus‐level identification of isolates was achieved by the 16S rRNA gene sequencing using the bacterial universal primers F27 (5′‐AGA GTT TGA TCM TGG CTC AG‐3′) and R1492 (5′‐TAC GGY TAC CTT GTT ACG ACT T‐3′) (Weisburg, Barns, Pelletier, & Lane, 1991). Reaction mixtures (25 μL) were prepared as follows: 8.75 μL of Ultrapure water, 5 μL of GoTaq^®^ Flexi DNA Polymerase Buffer, 3 μL of MgCl_2_ 25 mM, 2.5 μL dNTPs 2 mM, 1.5 μL of each primer 10 μM, 1.25 μL of DMSO 100%, 0.5 μL GoTaq^®^ Flexi DNA Polymerase 5 U/μL (Promega, Madison, WI), and 1 μL of DNA template. Thermal cycling started with an initial denaturation step of 94°C for 3 min, followed by 35 cycles of 94°C for 1 min, 55°C for 1 min, 72°C for 2 min, and a final extension step of 72°C for 10 min. PCR amplifications were carried out in a MyCycler^™^ Thermal Cycler System (Bio‐Rad, Berkeley, CA) and products were analyzed under UV light after electrophoresis in 1.5% agarose gels stained with RedSafe^™^ (iNtRON Biotechnology, INC, Korea). PCR was then purified with the DNA Clean & Concentrator^™^‐5 kit (Zymo Research Europe GmbH, Freiburg, Deutschland) and sequenced by the Sanger method using the BigDye Terminator 3 chemistry (Applied Biosystems, Foster City, California) using the same forward and reverse primers. Sequences were automatically trimmed using the Phred quality score method (Sorensen, Rasmussen, Eriksen, Larsen, & Morling, 2004). Their closest phylogenetic relatives were searched using the Basic Local Alignment Search Tool (BLAST) of the National Center for Biotechnology Information (NCBI) database (Zhang, Schwartz, Wagner, & Miller, 2000). Phylogenetic analysis of the isolates sequences and of the five blasts closest relatives was performed using MEGA software (Kumar, Stecher, & Tamura, 2016). Phylogenetic tree image was produced using the Dendroscope software (Huson et al., 2007).

### 2.3 Extraction procedures

Bacteria were grown in 1 L balloons with 0.5 L of Marine Broth at 20°C with constantly filtered aeration during 3 days. After that, bacteria cells were centrifuged and the lyophilized pellet was extracted during 12 hr with methanol:dichloromethane (1:1) solvents at constant stirring and protected from the light. The solvents were evaporated in a rotary evaporator and the extracts solubilized in dimethyl sulfoxide (DMSO) and stored at −20°C until further use.

### 2.4 Bioactivities of bacteria extracts

#### 2.4.1 Antimicrobial activities

Antimicrobial activity of bacteria extracts was evaluated against *Pseudomonas aeruginosa* (ATCC 27853), *Escherichia coli* (ATCC 25922), *Salmonella enteritidis* (ATCC 13076), and *Bacillus subtilis* (ATCC 6633) grown in Lysogeny broth (LB); *Staphylococcus aureus* (ATCC 25923) was grown in Trypticase Soy Yeast Extract medium (TSYE); *Candida albicans* (ATCC 10231) and *Saccharomyces cerevisiae* (ATCC 9763) were grown in Yeast Extract Peptone Dextrose (YPD) medium. All mediums were obtained from Merck (Darmstadt, Germany). The antimicrobial experiences were accomplished according to Horta et al. (2014). Bacteria were incubated at 37°C and fungi at 30°C. Ampicillin and bacitracin (Sigma Aldrich, Oakville, Canada) were used as positive controls (0.01–100 μg/mL). The antimicrobial activity was accompanied by optical density at 600 nm in order to verify the ability of extracts to inhibit the microorganisms growth (Synergy H1 Multi‐Mode Microplate Reader, BioTek^®^ Instruments, VT). For the most potent extracts (microorganism growth inhibition > 60%), the IC_50_ was determined (10–1,000 μg/mL). Results were expressed in percentage of growth inhibition relative to the control (growth medium with microorganism).

#### 2.4.2 Antitumor activities

##### Cell culture conditions

The antitumor activities were performed in two in vitro human cancer cell models, namely hepatocellular cancer cell line (HepG‐2 cells) (ATCC HB‐8065) and a human breast adenocarcinoma cell line (MCF‐7 cells) (ACC 115), previous acquired in American Type Culture Collection (ATCC) and DSMZ—German collection of microorganisms and cultured biobanks, respectively. Cancer cell lines were cultivated according to the information supplied by the biobanks. Cells medium was changed every 3 days and the cells reached confluence after 5–6 days of initial seeding. For subculture, the cells were dissociated with trypsin‐EDTA, split 1:3, and subculture in Petri dishes with 25 cm^2^ growth area. Cells were maintained at 37°C, 95% of humidity, and 5% CO_2_.

#### 2.4.3 Cytotoxic and antiproliferative activities

Cytotoxic and antiproliferative activities were performed according to Alves et al. (2016). Cancer cells were treated with bacteria extracts (1 mg/mL) for 24 hr and the effects were estimated using a colorimetric assay based on the conversion of tetrazolium dye (MTT) (Sigma, Germany) to a blue formazan product by live mitochondria after spectrophotometric reading at 570 nm (Mosmann, 1983). Cisplatin and tamoxifen were used as standard drugs (0.1–100 μg/mL; 24 hr) and their IC_50_ was determined.

### 2.5 Statistical analysis

One‐way analysis of variance (ANOVA) was performed followed by Dunnett's test to discriminate significant differences between bacteria extracts and control. These analyzes were performed with GraphPad InStat for Windows. Results are presented as mean ± standard error of the mean (SEM). The significance level was inferred at *p *<* *0.05. The IC_50_ concentration was calculated by the analysis of non‐linear regression by means of the equation: *Y* = 100/(1 + 10^(*X* − Log^ IC_50_^)^). Calculations were performed using GraphPad v5.1 (GraphPad Software, La Jolla, CA, USA) software.

## 3 RESULTS

### 3.1 Isolation and identification of macroalgae‐associated bacteria

**Figure 2:**
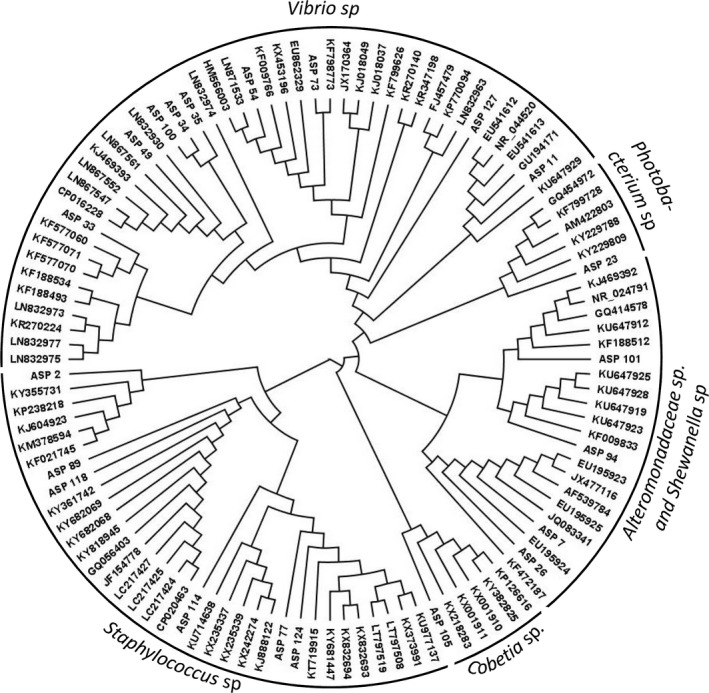
Phylogenetic tree based on the 16S rRNA gene sequences of *Asparagopsis armata*‐associated bacteria and the top five blasts best‐hit sequences identified using the NCBI blast program

In this study, 21 phenotypically different bacteria were isolated from *Asparagopsis armata* surface and analyzed. The 16S rDNA sequencing allowed to identify the isolates at the genera level (Table 1). The sequences have been deposited in GenBank (accession numbers MK408483–MK408503). Phylogenetic analysis of 16S rRNA gene sequences revealed that the bacteria isolated from *A. armata* belong to seven different genera: *Vibrio*,* Staphylococcus*,* Bacillus*,* Cobetia*,* Photobacterium*, and the two closely related *Shewanella* and *Alteromonadaceae* (Figure 2). The genus *Vibrio* was the most representative, with 9 (42.86%) isolates followed by *Staphylococcus* with 5 (23.80%).

**Table 1:** Genus identification of *Asparagopsis armata*‐associated bacteria obtained by 16S rRNA sequencing

Associated bacteria	Genus	Occurrence (%)
ASP 11; ASP 33; ASP 34; ASP 35; ASP 49; ASP 54; ASP 73; ASP 100; ASP 127	*Vibrio*	42.86
ASP 77; ASP 89; ASP 114; ASP 118; ASP 124	*Staphylococcus*	23.80
ASP 26; ASP 94; ASP 101	*Shewanella*	14.29
ASP 7	*Alteromonadaceae*	4.76
ASP 2	*Bacillus*	4.76
ASP 105	*Cobetia*	4.76
ASP 23	*Photobacterium*	4.76

### 3.2 Antimicrobial activity

**Figure 3:**
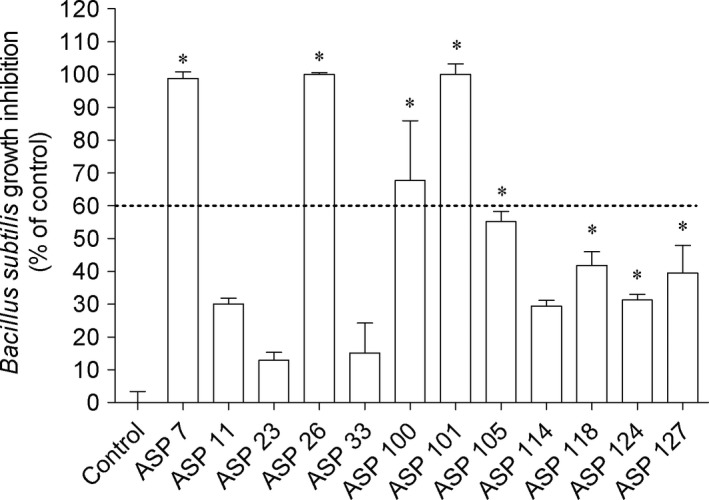
Associated bacteria extracts (1 mg/mL) that induced effects against *Bacillus subtilis* growth. Values correspond to average ± SEM (*n* = 4). Symbols represent statistically significant differences (One‐way ANOVA, Dunnett's test, *p* <  0.05) when compared to: *control

**Figure 4:**
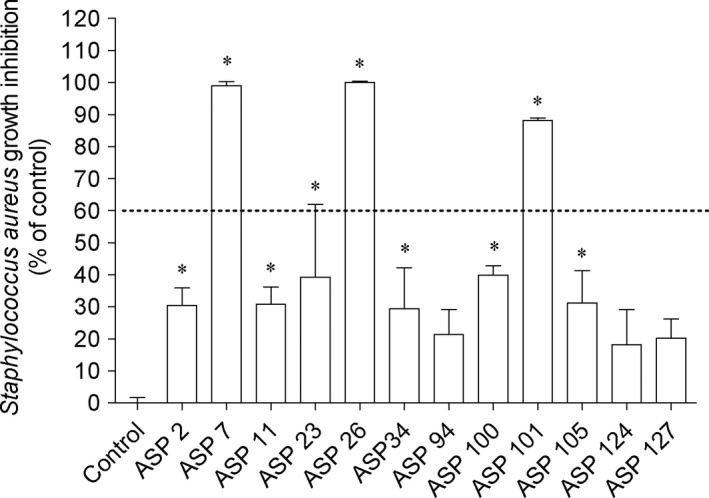
Associated bacteria extracts (1 mg/mL) that induced effects against *Staphylococcus aureus* growth. Values correspond to average ± SEM (*n* = 4). Symbols represent statistically significant differences (One‐way ANOVA, Dunnett's test, *p* <  0.05) when compared to: *control

In order to evaluate the antimicrobial potential of *A. armata*‐associated bacteria extracts, several microorganisms belonging to Gram+, Gram‐, and fungus were selected. Concerning the results, the highest antimicrobial activities of bacteria extracts were verified against *B. subtilis* and *S. aureus* existing statistical differences compared to control (Figures 3 and 4). The extracts did not display ability to inhibit the growth of the remaining microorganisms. In addition, among the bacteria extracts tested, ASP 7, ASP 26, and ASP 101 mediated the highest effect on both microorganisms. Those bacteria belong to the two closely related genus *Shewanella* and *Alteromonadaceae*. In addition, since those extracts reduced the microorganisms growth in more that of 60%, it was decided to determine their IC_50_ (Table 2). The extracts of Asp 26 (151.1 and 346.8 μg/mL) and Asp 7 (188.5 and 384.0 μg/mL) exhibited the smallest IC_50_ against *B. subtilis* and *S. aureus*, respectively.

**Table 2:** IC_50_ values obtained for associated bacteria extracts (10–1,000 μg/mL) that showed the highest inhibition (>60%) against *Staphylococcus aureus* and *Bacillus subtilis* as well as for standard drugs (0.01–100 μg/mL)

		IC_50_ μg/mL	
		*Staphylococcus aureus*	*Bacillus subtilis*
Bacteria extracts	ASP 7	384.0 (255.4–578.6)	188.5 (106.4–334.1)
	ASP 26	346.8 (216.4–555.8)	151.1 (85.7–266.1)
	ASP 100	—	1034.0 (488.9–2186.9)
	ASP 101	546.1 (357.6–834.1)	505.5 (267.7–954.6)
Standard drugs	Ampicillin	0.038 (0.029–0.051)	0.16 (0.12–0.21)
	Bacitracin	4.06 (3.36–4.90)	4.1 (3.30–5.07)

Values are expressed as means with 95% confidence intervals. At least three independent experiments were performed.

### 3.3 Antitumor activity

**Figure 5:**
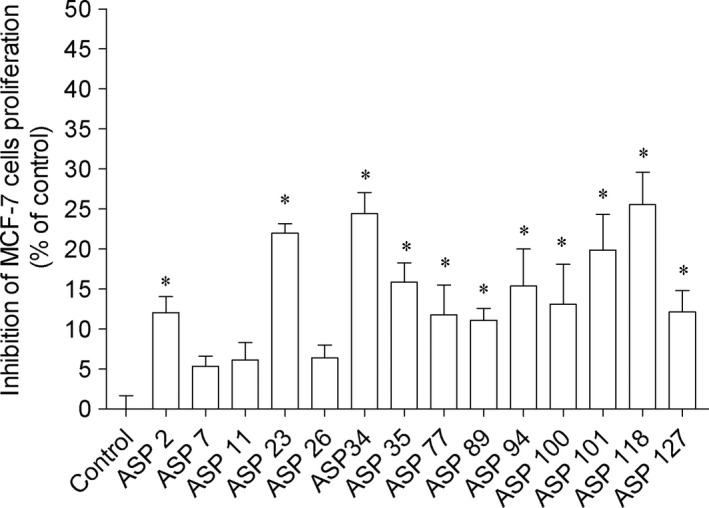
Associated bacteria extracts (1 mg/mL; 24 hr) that induced effects on MCF‐7 cells proliferation. Values correspond to average ± *SEM* (*n* = 4). Symbols represent statistically significant differences (One‐way ANOVA, Dunnett's test, *p* < 0.05) when compared to: *control. Tamoxifen exhibited an IC_50_ of 10.10 μg/mL

**Figure 6:**
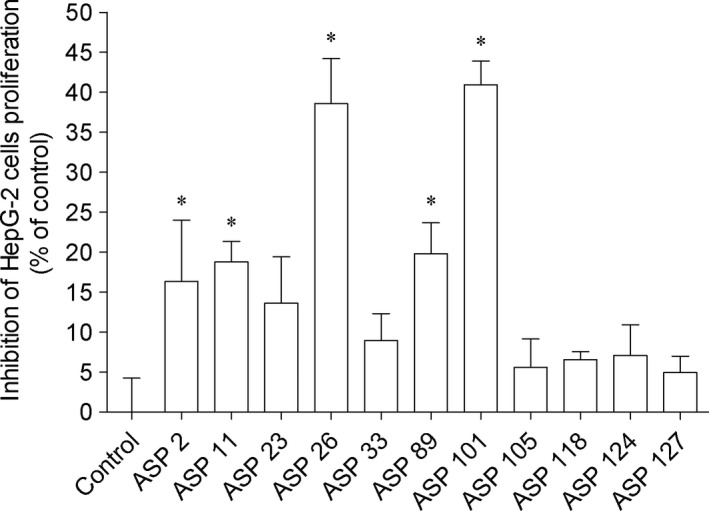
Associated bacteria extracts (1 mg/mL; 24 hr) that induced effects on HepG‐2 cells proliferation. Values correspond to average ± *SEM* (*n* = 4). Symbols represent statistically significant differences (One‐way ANOVA, Dunnett's test, *p* < 0.05) when compared to: *control. Cisplatin exhibited an IC_50_ of 22.63 μg/mL

Regarding the antitumor potential of bacteria extracts, those did not induced cytotoxicity on MCF‐7 and HepG‐2 cells (data not shown). MCF‐7 and HepG‐2 are cell lines frequently used for the screening of compounds to detect potential anticancer activity. These cell lines derived from two of the most deadly or aggressive cancers, breast cancer and liver cancer, respectively. In the cell proliferation studies, several bacteria extract displayed ability to reduce the proliferation of MCF‐7 and HepG‐2 cells (Figures 5 and 6, respectively) exhibiting significant differences compared to control. Among them, the highest activity was mediated by extracts derived from ASP 26 and Asp 101 bacteria that reduced HepG‐2 cells proliferation around 40% (1 mg/mL; 24 hr) (Figure 6). The positive controls cisplatin and tamoxifen when tested on HepG‐2 and MCF‐7 cells exhibited an IC_50_ of 22.63 μg/mL (18.24–28.07) and 10.10 μg/mL (8.40–12.14), respectively.

## 4 DISCUSSION

In the marine environment, bacteria found associated with macroorganisms have been recognized to be an excellent source of bioactive compounds. In fact, these marine macroorganisms can ultimately be a sustainable solution to obtain bioactive metabolites with socioeconomic relevance in several industries, including pharmaceutical industry (Murray et al., 2013). In previous works, bacteria associated with macroorganisms such as sponges and seaweeds have been identified as important producers of new bioactive compounds with therapeutic potential, including antimicrobial and antitumor activities (Alvarado, Huang, Wang, Garrido, & Leiva, 2018; Ibrahim et al., 2018; Imamura et al., 1997; Kanagasabhapathy, Sasaki, & Nagata, 2008; Kuo et al., 2019). Seaweeds belonging to the Bonnemaisoniaceae family, such as *A. armata*, have shown to produce potent secondary metabolites with antibacterial properties which can be involved in the biofilms microbiota regulation (Nylund, Cervin, Hermansson, & Pavia, 2005; Pinteus et al., 2015, 2018; Rhimou, Hassane, José, & Nathalie, 2010).

In this context, the present work was conducted in order to identify the bacterial community associated with the red seaweed *Asparagopsis armata* and investigate their potential as producers of antimicrobial and antitumor substances. Analyzing the results, it was possible to verify that *Vibrio* was the most abundant genera with an occurrence of approximately 43% (Table 1). In agreement with these results, Mahmud et al. (2008, 2007) also identified *Vibrio* as dominant epiphytic species in seaweeds studied. Moreover, Gallardo, Risso, Fajardo, and Belchior (2004) also demonstrated that *Vibrio* and *Staphylococcus* were the predominant genera associated with green seaweed *Monostroma undulatum*. In fact, *Vibrio* species are widely common in aquatic environments, especially in coastal waters, which may justify their high occurrence in aquatic samples. However, different results were obtained in a study of the bacterial communities associated with *A. armata* in three locations in the southwest coast of Portugal, using a metagenomic approach (Aires, Serrão, & Engelen, 2016) in which considering the samples identified to the family level less than 1% belongs to the Vibrionaceae family. The second most frequent family observed in our study *Staphylococcaceae* (23.8%) was not detected in the study of Aires and coworkers, in which the most frequent bacteria belong to the *Saprospiraceae* family (between 36% and 47% depending on the location) that was not detected by us. Despite the different methods used, the differences observed reinforce the role of geographical localization and environmental conditions in the bacteria community diversity associated to algae (Aires et al., 2016).

Concerning the antimicrobial potential of bacteria extracts, the strongest effects were exhibited by an *Alteromonadaceae* (ASP 7) and a *Shewanella* strain (ASP 26) against *S. aureus* and *B. subtilis* being more marked against *B. subtilis. Alteromonadaceae* and *Shewanella* belong to the γ‐proteobacteria family, which are frequently found associated with marine invertebrates such as seaweeds and sponges (Thomas, Kavlekar, & LokaBharathi, 2010 and references there in). In addition, an important work developed in 2016 by a Portuguese team revealed the main bacterial families associated with *Asparagopsis armata* collected and also verified the presence of the γ‐proteobacteria family; however, they did not test their bioactive potential (Aires et al., 2016). In agreement with our results, several works investigated the bioactive potential of these group of bacteria and results revealed potential to produce secondary metabolites with high antimicrobial potential (Thomas et al., 2010 and references in; El Bour, Ismail‐Ben Ali, & Ktari, 2013 and references in). Lemos, Toranzo, and Barja (1985) evaluated the antimicrobial activity of 224 bacteria isolated from green and brown algae, and 38 displayed antimicrobial activity, including against *Staphylococcus aureus*. Hamid, Usup, and Ahmad (2013) isolated 494 bacteria from different marine sources and almost half presented antimicrobial activity against several organisms, including *Staphylococcus aureus* and *Bacillus subtilis*. These secondary metabolites are crucial for bacterial–host interactions, especially by mediating surface biofilms communities (Thomas et al., 2010 and references in; Wilson, Raftos, & Nair, 2011), therefore preventing unfavorable competing species to establish, such as the widely abundant *Staphylococcus* and *Bacillus* strains (Gunn & Colwell, 1983; Wilson et al., 2011). Another interesting work was recently conducted to understand the effects of seaweeds addition to animal feed, and interestingly, it was verified that the methane production decreased substantially with the addition of *Asparagopsis* species to the feed, which can be associated with the high antimicrobial potential of these seaweed family and their associated bacteria (Machado, Magnusson, Paul, de Nys, & Tomkins, 2014).

Interestingly, in the present work, the antimicrobial activities induced by bacteria extracts were only observed against Gram positive (+) bacteria. Accordingly, other studies on marine invertebrates associated bacteria also shown the highest antibacterial activity against Gram (+) bacteria (Anand et al., 2006; Boyd, Adams, & Burgess, 1999; Villarreal‐Gomez et al., 2010). In fact, the generally low activity of the extracts against the Gram‐negative (−) microorganisms can be related with the presence of an outer membrane and a periplasmic space with defensive enzymes in Gram (−) bacteria, which are absent in Gram (+) bacteria. These are effective barriers against foreign substances, such as antimicrobial compounds (Lambert, 2002; Sofidiya, Odukoya, Afolayan, & Familoni, 2009). Concerning the low antifungal activity, fungi are characterized by having an individualized nucleus (eukaryotic) which itself is an effective barrier to antimicrobial drugs (Azevedo et al., 2015).

Bacteria associated with marine invertebrates have also been an interesting target in the search of new compounds with antitumor potential. For instance, violacein derived from bacterial biofilms exhibited the ability to inhibit protozoan cells and promotes apoptosis in eukaryotic cells. Seaweed‐associated bacteria belonging to Firmicutes, Proteobacteria, and Actinobacteria families exhibited the ability to reduce cell viability of HCT‐116 colorectal cancer cells (Villarreal‐Gomez et al., 2010). In the present study, the antitumor potential of associated bacteria extracts was evaluated on two different human cancer cells lines, HepG‐2 and MCF‐7 cells, through cytotoxicity and antiproliferative assays. The results obtained in this study suggest that bacteria extracts contain compounds that mediate selective and differentiating effects between hepatocellular and breast cancer cells lines. Interestingly, two extracts from *Shewanella* strains (Asp 26 and Asp 101) exhibited antiproliferative activity on HepG‐2 cells for nontoxic concentrations suggesting that the effects observed can be mediated by the presence of compounds with cytostatic activity, which can directly affect the cell cycle. In fact, the essential processes of cell proliferation are finely regulated by the cell cycle and apoptosis, which is mediated through the interaction of various families of proteins. These processes are distinct, but closely related, as evidenced by the central role of p53 in the cell cycle and apoptosis processes (Sa & Das, 2008). Thus, in further studies, it will be important to analyze the cell cycle regulation to address this aspect. Comparing with the standard drugs, the effects mediated by ampicillin, bacitracin, cisplatin, and tamoxifen were higher than the effects mediated by bacteria extracts. This is not surprising since the bacteria extracts examined in this work are complex mixtures of several compounds, which may contain a low concentration of the active compounds, and consequently, their activity may be enhanced by isolating the compounds linked to the antimicrobial and antitumor activities. In contrast, standard drugs are mainly composed by the active compound.

In this work, it was possible to verify that bacteria associated with *Asparagopsis armata* present antimicrobial activity against Gram (+) microorganisms. Moreover, some bacteria also revealed to produce substances with antiproliferative effects at nontoxic concentrations. Altogether, the results presented in the present work allowed to disclosure the cultivable *A. armata* microbiological community. In addition, the most promising bacteria for the isolation and purification of antimicrobial and antitumor substances were identified, opening a new window of opportunities for the sustainable production of biotechnologically relevant structures.

## CONFLICT OF INTERESTS

The authors declare no conflict of interest.

## AUTHORS CONTRIBUTION

AH and CA developed the main experiments (seaweed collection, bacteria growth, isolation and identification, production of bacteria extracts, and bioactivity assays). JF and DR have been involved in bacteria growth and extraction procedures. CL, JR and NF have been involved in bacteria identification. SP and JS have been involved in the development of biological assays. AR designed and coordinated the bacteria identification process and RP designed, coordinated, and conceived the study. All authors contributed in the writing of the manuscript.

## ETHICS STATEMENT

None required.

## Data Availability

All data are provided in full in the results section of this paper apart from the 16S rRNA gene sequencing of the genera identified. The sequences have been deposited in GenBank (accession numbers MK408483–MK408503).
